# Gemcitabine/Nab-Paclitaxel versus FOLFIRINOX in Locally Advanced Pancreatic Cancer: A European Multicenter Study

**DOI:** 10.3390/cancers13112797

**Published:** 2021-06-04

**Authors:** Nicolas Williet, Angelica Petrillo, Gaël Roth, Michele Ghidini, Mila Petrova, Julien Forestier, Anthony Lopez, Audrey Thoor, Lucie Weislinger, Ferdinando De Vita, Julien Taieb, Jean Marc Phelip

**Affiliations:** 1 Department of Hepatogastroenterology, University Hospital of Saint-Etienne, 42000 Saint-Etienne, France; j.marc.phelip@chu-st-etienne.fr; 2 Department of Precision Medecine, University of Study of Campania «L. Vanvitelli», 81100 Naples, Italy; angelic.petrillo@gmail.com (A.P.); ferdinando.devita@unicampania.it (F.D.V.); 3 Hepato-Gastroenterology Department, University Hospital of Grenoble, 38043 Grenoble, France; groth@chu-grenoble.fr (G.R.); athoor@chu-grenoble.fr (A.T.); 4 Department of Medical Oncology, Cancer Center, Hospital of Cremona, 26100 Cremona, Italy; michele.ghidini@policlinico.mi.it; 5 Department of Medical Oncology, MHAT Nadezhda, 1220 Sofia, Bulgaria; milllapetrova@gmail.com; 6 Department of Medical Oncology, Hôpital Edouard Herriot, 69622 Lyon, France; julien.forestier@chu-lyon.fr; 7 Hepato-Gastroenterology Department, University Hospital of Nancy, 54500 Vandoeuvre-lès-Nancy, France; anthony-lopez@hotmail.fr (A.L.); lucieweislinger@yahoo.fr (L.W.); 8 Department of Gastroenterology and Gastro-Intestinal Oncology, Hôpital Européen Georges-Pompidou, APHP, Paris Descartes University, Sorbonne Paris Cité, 75004 Paris, France; jtaieb75@gmail.com

**Keywords:** pancreatic cancer, FOLFIRINOX, gemcitabine/nab-paclitaxel, prognostic factors, predictors of response

## Abstract

**Simple Summary:**

Gemcitabine/nab-paclitaxel (GN) and FOLFIRINOX (FFX) are two standard first-line therapies for metastatic pancreatic cancer (PC) but have rarely been compared, especially in patients with locally advanced PC (LAPC). By carefully selecting patients, it is likely these two regimens lead to similar survival outcomes. Through a multicenter European study, biases regarding practice habits are reduced. Hence, we observed no difference between GN and FFX as first-line treatments in patients with LAPC in terms of either survival, tumor response or tumor resection rate. Further trials are needed to confirm these data.

**Abstract:**

Background: Gemcitabine/nab-paclitaxel (GN) and FOLFIRINOX (FFX) are two standard first-line therapies for metastatic pancreatic cancer (PC) but have rarely been compared, especially in patients with locally advanced PC (LAPC). Methods: This is a retrospective European multicenter study including patients with LAPC treated with either GN or FFX as the first-line therapy between 2010 and 2019. Coprimary objectives were progression-free survival (PFS) and overall survival (OS), both estimated using the Kaplan–Meier method. Results: A total of 147 patients (GN: *n* = 60; FFX: *n* = 87) were included. Tumor resection rates were similar between the two groups (16.7% vs. 16.1%; *p* = 1), with similar R0 resection rates (88.9%). Median PFS rates were not statistically different: 9 months (95% CI: 8–13.5) vs. 12.1 months (95% CI: 10.1–14.6; *p* = 0.8), respectively. Median OS rates were 15.7 months (95% CI: 12.6–20.2) and 16.7 months (95% CI: 14.8–20.4; *p* = 0.7), respectively. Abdominal pain at the baseline (HR = 2.03, *p* = 0.03), tumors located in the tail of the pancreas (HR = 4.35, *p* = 0.01), CA19-9 > 200 UI/L (HR = 2.03, *p* = 0.004) and tumor resection (HR = 0.37, *p* = 0.007) were independent prognostic factors for PFS, similarly to OS. CA19-9 ≤ 200 UI/L (OR = 2.6, *p* = 0.047) was predictive of the tumor response. Consolidation chemoradiotherapy, more often used in the FFX group (11.7% vs. 50.6%; *p* < 0.001), was not predictive. Conclusion: This retrospective study did not show any difference between GN and FFX as the first-line treatment in patients with LAPC.

## 1. Introduction

Pancreatic ductal adenocarcinoma (PDAC) is becoming the second cause of cancer-related deaths in Europe and America [1,2]. About one third of patients have a locally advanced disease at diagnosis. There is no standard first-line therapy in this setting except for gemcitabine [3,4,5]. Indeed, in randomized clinical trials (RCTs) carried out before 2009, metastatic and locally advanced PDAC were identified as advanced diseases. Survival outcomes and evaluation of treatment benefits were issued on the basis of subgroup analyses [6,7]. Hence, similarly to the metastatic setting, no superiority was observed when using a combination of chemotherapy with gemcitabine compared to gemcitabine alone in LAPC [6,7]. Since 2009, it has been recommended to separate the metastatic and locally advanced settings in RCTs for PDAC [8,9]. However, there is still a lack of dedicated phase III RCTs on LAPC and no positive or practice-changing results have been reported in this field. Hence, patients with LAPC tend to be treated similarly to those in the metastatic setting by using FOLFIRINOX (fluorouracil, leucovorin, oxaliplatin and irinotecan) (FFX) or gemcitabine plus nanoparticle albumin-bound (nab)-paclitaxel (GN), both standard first-line therapies in select patients [10,11]. Numerous retrospective and some prospective studies conducted on the LAPC treated using these regimens have shown survival outcomes that appear to be clearly better than those observed in studies evaluating gemcitabine alone [6,7,12,13,14,15]. Moreover, according to meta-analyses and some prospective studies, high response rates related to these chemotherapy regimens lead to secondary surgery in 15–28% of cases in LAPC [14,15,16,17,18] compared to 5–10% under gemcitabine. For these reasons, FFX and GN are highly recommended for borderline resectable tumors and are an option for LAPC [3,4,5]. However, there is still a lack of evidence regarding a direct comparison of these two chemotherapy regimens in LAPC. Based on this background, this European multicenter study aimed to evaluate survival outcomes in patients with LAPC treated either with GN or FFX in a real-life setting.

## 2. Materials and Methods

### 2.1. Study Population

This is a retro-prospective European study involving centers from France (*n* = 4), Italy (*n* = 2) and Bulgaria (*n* = 1). All the patients consecutively treated for LAPC using either FFX or GN as the first-line therapy between 2010 and 2018 were included. The patients included from January 2018 were prospectively followed to December 2019. The other inclusion criteria were diagnosis of previously untreated LAPC according to the 2017 National Comprehensive Cancer Network (NCCN) guidelines [19]. The exclusion criteria were distant metastases and anatomically resectable or borderline resectable tumors. Demographic and clinical details were collected. Baseline cross-sectional imaging was reviewed and definition of locally advanced disease was based on the NCCN classification. Histological or cytological diagnosis was obtained for each patient before treatment delivery. Institutional review board consent was obtained for each center. All the patients provided written informed consent (at the time of therapy) about the use of their data for future medical research.

### 2.2. Chemotherapy Regimens

Nab-paclitaxel (125 mg/m^2^) followed by gemcitabine (1000 mg/m^2^) was administered intravenously on days 1, 8 and 15 every four weeks. The patients who were treated with FOLFIRINOX received oxaliplatin (85 mg/m^2^), irinotecan (150–180 mg/m^2^), leucovorin (400 mg/m^2^) and 5-fluorouracil (5-FU) (bolus (0 to 400 mg/m^2^) + intravenous infusion (2400 mg/m^2^ for 46–48 h)). All the patients were provided the first-line therapy until progression of the disease, unacceptable toxicity or patient refusal. However, after 4–6 months of chemotherapy without progression of the disease, some of the patients underwent either consolidation chemoradiotherapy (CCR), continuation treatment, maintenance therapy (with capecitabine or LV5FU2 for FFX or gemcitabine alone for GN) or a therapeutic break at the clinician’s discretion. Chemotherapy-related toxicity was graded according to the National Cancer Institute’s Common Terminology Criteria for Adverse Events (NCI CTCAE version 4.0). Modifications of the chemotherapy regimen (dose reductions) were left at the clinician’s discretion. Generally, antiemetic prophylaxis with serotonin type 3 receptor antagonists plus dexamethasone was used. Recombinant human granulocyte colony-stimulating factor (G-CSF) and erythropoietin were administered as needed by the physician.

### 2.3. Outcome Evaluation

The primary endpoints were progression-free survival (PFS) and overall survival (OS) which were defined from the date of the first course of chemotherapy to the date of the event (disease progression or death, respectively) or to the last follow-up. In case of any event, the patients were censored at the time of last-follow-up. Disease progression and overall tumor response were assessed using the Response Evaluation Criteria in Solid Tumors (RECIST) version 1.1 [20]. The secondary endpoints were the secondary surgery rate, overall response rate according to the RECIST criteria, therapeutic strategies in case of overall disease control (CCR, continuation treatment, maintenance treatment, therapeutic break) and chemotherapy-related toxicity. In case of a secondary surgery, the date of disease progression was considered as the date of disease recurrence.

### 2.4. Statistical Analyses

All statistical analyses were performed using R^®^ version 3.2.2 (R project, Auckland, New Zealand). The survival rates were estimated using the Kaplan–Meier method. Prognostic factors and predictors of tumor response and secondary surgery were identified using the Cox proportional hazards regression. Multivariate analyses were performed by selecting variables according to their corresponding *p*-value (≤0.05) in univariate analyses and by adjusting for prognostic covariables which would be unbalanced between the two groups at the baseline (especially age and performance status according to the Eastern Cooperative Oncology Group scale (ECOG)). Quantitative variables were reported as the medians with the corresponding interquartile range and compared using the Wilcoxon test. Qualitative variables were reported as numbers and percentages and compared using the chi-squared or Fisher’s test.

## 3. Results

### 3.1. Study Population

Of the 172 patients initially enrolled, 25 were excluded after reviewing the exclusion criteria (metastatic disease: *n* = 1; borderline resectable tumors: *n* = 13; locally advanced recurrence: *n* = 9; unknown stage of the disease: *n* = 2). Hence, a total of 147 patients (GN: *n* = 60; FFX: *n* = 87) were finally included (France: 11–31 patients/center; Italy: 15–22/center; Bulgaria: *n* = 12). The main patients’ characteristics are reported in Table 1. The patients were 66 years old (interquartile ranges (IRQ): 59–72), with the sex ratio of 0.56 in favor of men and a history of diabetes or arterial hypertension in about one third of cases. At the baseline, 133 (95%) had performance status (PS) 0 or 1 according the ECOG scale. The primary tumor location was mainly in the head of the pancreas (72.1%). The median serum bilirubin, albumin and CA19-9 levels were 12 µmol/L (6.5–19), 36.6 g/L (33–40) and 127 UI/L (36.1–471), respectively. The patients constituting the GN and FFX groups differed regarding age (median: 71 vs. 63 years, *p* < 0.001; >70 years: 55% vs. 13.8%, *p* < 0.001), ECOG PS 0/1 (87.7% vs. 100%; *p* = 0.001) and CA19-9 (median: 83 UI/L vs. 172 UI/L; *p* = 0.07). All these factors were used as the adjustment for multivariate analyses. FFX was mostly prescribed in France (74.7%). Patients treated with GN were from France (55%) and Italy (45%). All the patients from Bulgaria were treated with FFX (*n* = 12).

### 3.2. Therapeutic Sequence and Events Potentially Impacting Survival

The median number of chemotherapy courses in the GN and FFX groups was 12 (IQR: 9–18) and 8 (IQR: 5–10) (*p* < 0.001), respectively. Tumor response according to the RECIST criteria was similar in the two groups (21.7% vs. 20.7%; *p* = 1). Consolidation chemoradiotherapy was performed in 11.7% and 50.6% (*p* < 0.001) of the patients, respectively. Secondary surgery was possible in ~16% in both groups (*p* = 1) (duodenopancreatectomy: 80.0% vs. 78.6%; *p* = 1) with similar pathological results regarding tumor (*p* = 0.81), nodes (*p* = 0.49) and negative resection margin (R0) rates (~89%; *p* = 1).

High-grade toxicity related to chemotherapy was observed in 26.7% vs. 28.4% (*p* = 0.97) of the patients, respectively. There was a numericall difference in favor of FFX regarding anemia (6.7% vs. 0.0%; *p* = 0.054) and neutropenia (18.3% vs. 6.9%; *p* = 0.062) in contrast with digestive toxicity (5.0% vs. 5.7%). In case of high-grade toxicity, the next course was delayed with a reduced dose in the vast majority of cases. Only six patients treated with FFX experienced discontinuation of the treatment for toxicity. A minority of the patients experienced too many alterations to switch to the second-line therapy in case of early disease progression (11.7% vs. 1.1%; *p* = 1).

During the follow-up, second-line chemotherapy was possible in 48.3% vs. 41.1% (*p* = 0.51) of the patients, respectively. FFX as the first line followed by GN as the second line was observed in 12.6% of the patients. The reverse sequence was possible in 3.3% in addition to 5% of the patients who underwent LV5FU2 + nanoliposomal irinotecan (nal-IRI) as the second-line therapy (Table 2). Third-line chemotherapy was reported in 21.7% vs. 6.9% (*p* = 0.02) of the patients, respectively.

### 3.3. Survival and Prognostic Factors

**Figure 1:**
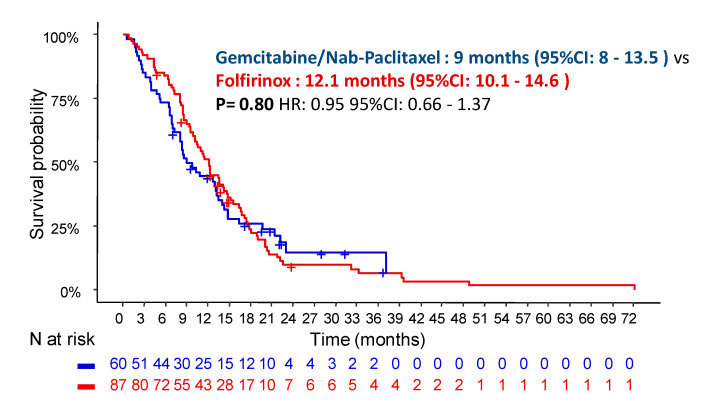
Progression-free survival depending on the chemotherapy regimen in patients with locally advanced pancreatic cancer.

The median follow-up was 15.2 months (95% confidence interval (CI): 9.4–21.9). The rates of events were 90.8% vs. 80.0% for disease progression and 85.1% vs. 73.3% for death, respectively. There was no statistical difference in terms of the PFS between patients treated with GN (9 months; 95% CI: 8–13.5) and FFX (12.1 months; 95% CI: 10.1–14.6; *p* = 0.80) (Figure 1). 

In univariate analyses, abdominal pain at the baseline (*p* = 0.027; hazard ratio (HR) = 1.8; 95% CI: 1.07–3.02), tumor located in the tail of the pancreas (vs. the head: HR = 2.63, *p* = 0.016; vs. the body: HR = 3.03, *p* = 0.011), CA19-9 > 200 UI/L (HR = 1.79; *p* < 0.001) and secondary surgery (HR = 0.52; *p* = 0.011) were predictors of the PFS. After multivariate analyses, these factors remained significant independently of age (>70 years vs. ≤70 years: *p* = 0.12) and ECOG PS (0 vs. 1: *p* = 0.14; 0 vs. 2: *p* = 0.24). FFX was not superior to GN in terms of the PFS (*p* = 0.5) (Table 3).

**Figure 2:**
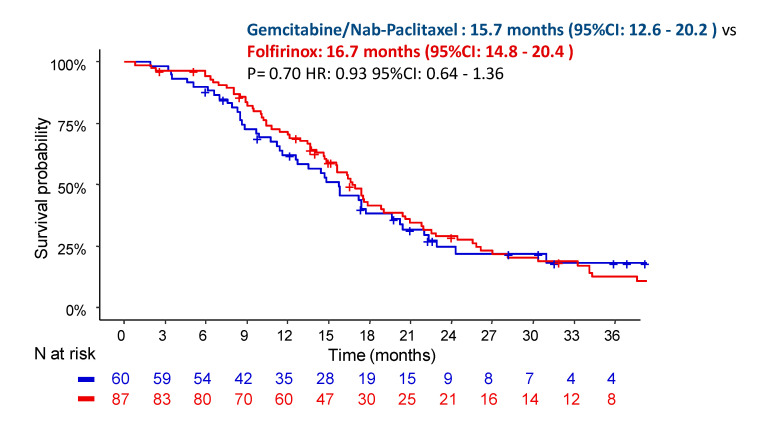
Overall survival depending on the chemotherapy regimen in patients with locally advanced pancreatic cancer.

There was no difference in terms of the OS between the patients treated with GN (median OS: 15.7 months; 95% CI: 12.6–20.2) and FFX (median OS: 16.7 months; 95% CI: 14.8–20.4; *p* = 0.70; HR = 0.93) (Figure 2). 

In the univariate analyses, ECOG PS 2 vs. PS 0 (HR = 2.68; *p* = 0.023), tumor location (tail vs. head: HR = 2.38, *p* = 0.042; tail vs. body: HR = 2.63, *p* = 0.033), CA19-9 > 200 UI/L (HR = 1.8; *p* < 0.001) and secondary surgery (HR = 0.4; *p* = 0.002) were predictive. After multivariate analyses, these factors remained predictive independently of age (>70 years vs. ≤70 years: *p* = 0.92), ECOG PS (PS 1 vs. PS 0: *p* = 0.21, PS 2 vs. PS 0: *p* = 0.47) and the chemotherapy regimen (*p* = 0.60) (Table 4).

### 3.4. Predictors of Tumor Response and Secondary Surgery

Tumor response was observed in 15 (25%) patients treated with GN and in 20 (23%) patients treated with FFX (*p* = 0.99). In the univariate analyses, only CA19-9 ≤ 200 UI/L (*p* = 0.047; OR = 2.6; 95% CI: 1.05–7.1) was associated with tumor response. Neither the chemotherapy regimen (*p* = 0.84) nor consolidation chemoradiotherapy (*p* = 0.11) were associated with tumor response.

Secondary surgery was reached in 10 (16.7%) patients treated with GN and in 14 (16.1%) patients treated with FFX (*p* = 1). In the univariate analyses, CA19-9 ≤ 200 UI/L (*p* = 0.015; OR = 4.15; 95% CI: 1.44–15.05) was associated with secondary surgery, whereas tumor size < 40 mm (*p* = 0.051; OR = 2.99; 95% CI: 1.05–9.86) showed a trend towards this. Neither the chemotherapy regimen (*p* = 0.93) nor consolidation chemoradiotherapy (*p* = 0.99) were associated with tumor response.

## 4. Discussion

There is increasing evidence on FFX and GN as the first-line therapy in patients with LAPC [12,13,14,15,18,21,22,23,24,25,26,27,28,29,30,31]. Some of these studies are descriptive and show a median PFS of 8–12 months [12,13,18,29,30,31] and median OS of 18–24 months [12,13,18,25,26,27,28,29,30,31] either with FFX or GN, which is in accordance with the results of this study. However, the number of studies that compared survival outcomes between these two chemotherapy regimens in this setting is low [21,22,23,24,25,26,27,28,29,30,31]. Additionally, the majority of them included not only LAPC, but also borderline resectable tumors [23,27,29,31] or metastatic disease [21,22,24,25,26,28], resulting in a very heterogeneous study. 

In this study, we aimed to compare these two standard chemotherapy regimens only in patients with LAPC. However, as the other retrospective series, groups of patients were unbalanced regarding age and PS so that GN was globally reserved for older and more fragile patients. Indeed, nab-paclitaxel is not reimbursed both in France and in Bulgaria. It was prescribed by each French center (*n* = 4) in this study. Nab-paclitaxel is more expensive than FFX even after adjusting for the costs of G-CSF often used with FFX for the prevention of febrile neutropenia. Hence, in the French centers involved into this study, the use of GN (*n* = 33) depended on the patient’s age, ECOG PS and comorbidities. Despite this potential selection bias, FFX was not found superior to GN in terms of survival outcomes in our study. One of the reasons is that nab-paclitaxel is reimbursed in some countries such as Italy, leading to the use of GN as the preferentially prescribed first-line therapy due to its better toxicity profile and its non-reimbursement as the second-line treatment [32,33]. Italian patients treated with GN represented almost half of our cohort (*n* = 27/60) and allowed decreasing this bias. Moreover, treatment effects were compared in a multivariate model adjusting for age > 70 years and performance status according to the ECOG scale. Our study was not designed for matched population-based analyses. However, despite the use of statistical matching of the population, the risk of intrinsic biases was not null, as supported by controversial data, especially in the metastatic setting [28,30,34]. Randomized clinical trials are needed to obtain a prospective comparison between these two chemotherapy regimens. To the best of our knowledge, there are only two ongoing randomized clinical trials comparing GN and FFX in LAPC [35,36].

Additionally to survival outcomes, we showed no difference in terms of pancreatic resection rates (~16%), tumor response (~21%) and postoperative pathological examination results (pT, pN, R0) between the patients treated with GN or FFX. These results are in accordance with the prospective observational study LAPACT for GN [18] (secondary surgery: 15%) and with the recently updated large retrospective cohort of the AGEO study for FFX (18%) [15]. Presence of nodes found in the postoperative pathological examination seemed to be numerically greater in the patients treated with GN, but our study was not designed to evaluate the prognostic value of such findings due to the two very small corresponding subgroups (GN: *n* = 10; FFX: *n* = 14). Similarly, the negative resection margin (R0) rates were similar between the two groups (~89%; *p* = 1), which is in accordance with the recent noncomparative randomized phase II SWOG S1505 study that has evaluated the feasibility of intensive chemotherapy (GN or FFX) before and after surgery for resectable pancreatic cancer (R0: ~85%) [37]. 

Beyond the efficacy, the choice between these two chemotherapy regimens should be based on the safety profile and the quality of life. According to other retrospective studies, this toxicity was not different from the one determined in the PRODIGE-4 and MPACT trials [22]. Our controversial results regarding safety (similar rates of high-grade toxicity between the two regimens; low rate of digestive toxicity with FFX) could be explained by the treatment management by clinicians in real life. For example, the low grade 3–4 neutropenia observed in the FFX group (6.9%) compared to the GN group (18.3%) could be explained by the common prescription of G-CSF for the prevention of febrile neutropenia under FFX. Additionally, the modified FOLFIRINOX regimen (no 5-FU bolus + reduction of the irinotecan dose to 150 mg/m^2^ at the baseline) was largely prescribed in this cohort, and many more patients underwent CCR before a therapeutic break in the FFX group (11.7% vs. 50.6%; *p* < 0.01) which probably contributed to improving the hematologic as well as digestive toxicity. 

Finally, it is important to note the difficulty of taking into account all the optional strategies in LAPC during the first-line therapy such as consolidation chemoradiotherapy, maintenance treatment, therapeutic break or continuation of treatment until disease progression, which could potentially impact survival outcomes. There is still no standard strategy during the first-line therapy in LAPC. Consolidation chemoradiotherapy is a therapeutic option in LAPC for which the phase III LAP-007 trial failed to demonstrate a benefit regarding survival after the induction period with gemcitabine alone [38]. Maintenance treatment with LV5FU2 (or capecitabine) seemed not to be inferior to the continuous FFX regimen in the phase II Panoptimox trial in the metastatic setting [39]. However, such a strategy has not been evaluated in LAPC despite some clinicians applying it routinely. To the best of our knowledge, this is the first time these different strategies according to the baseline chemotherapy regimen were described and compared, even if no conclusion can be made regarding the best one of them. Additionally, GN as the first-line therapy can potentially offer more possibilities in the later lines of treatment. However, FFX as the first line followed by GN as the second line appeared more feasible than the inverse sequence in our study and as previously reported [40]. 

## 5. Conclusions

Despite all those limitations, our study showed no difference in terms of survival outcomes, pancreatic resection rates and tumor response between the patients treated with GN or FFX. Therefore, GN and FFX might represent alternative choices in the first-line treatment in patients with LAPC according to the clinician’s discretion, as well as the age and ECOG PS of patients. However, further prospective trials and RCTs are needed in order to confirm and validate these data in LAPC.

## Figures and Tables

**Table 1:** Patients’ characteristics.

Variables	Study Population	Gemcitabine/Nab-Paclitaxel	FOLFIRINOX	*p*-Value
	(*n* = 147)	(*n* = 60)	(*n* = 87)	
Country				<0.001
France (*n* = 4)	98 (66.7%)	33 (55%)	65 (74.7%)	
Italy (*n* = 2)	37 (25.2%)	27 (45%)	10 (11.5%)	
Bulgaria (*n* = 1)	12 (8.2%)	0 (0%)	12 (13.8%)	
Age, median (IQR)	66 (59–72)	71 (63.8–77)	63 (56–67.5)	<0.001
Age > 70 years, *n* (%)	45 (30.6%)	33 (55%)	12 (13.8%)	<0.001
Male sex, *n* (%)	83 (56.5%)	31 (51.7%)	52 (59.8%)	0.42
Arterial hypertension, *n* (%)	51 (34.7%)	15 (25%)	36 (41.4%)	0.48
Diabetes, *n* (%)	49 (33.3%)	24 (40%)	25 (28.7%)	0.21
History of myocardial ischemia, *n* (%)	11 (7.5%)	5 (8.3%)	6 (6.9%)	0.2
Performance status, *n* (%)				
ECOG 0–1	133 (95%)	50 (87.7%)	83 (100%)	0.001
ECOG 0	73 (49.7%)	21 (35%)	52 (59.8%)	<0.001
ECOG 1	60 (40.8%)	29 (48.3%)	31 (35.6%)	
ECOG 2	7 (4.8%)	7 (11.7%)	0 (0%)	
Missing data	7 (4.8%)	3 (5%)	4 (4.6%)	
Tumor location, *n* (%)				
Head	106 (72.1%)	38 (63.3%)	68 (78.2%)	0.11
Body	34 (23.1%)	19 (31.7%)	15 (17.2%)	
Tail	7 (4.8%)	3 (5%)	4 (4.6%)	
Bilirubin, median (IQR)	12 (6.5–19)	8.4 (1.2–14)	15 (9–20.8)	-
Albumin, median (IQR)	36.6 (33–40)	36.8 (35–40.9)	36 (33–39.5)	0.34
CA19-9, median (IQR)	127 (36.1–471.2)	83 (18–403.2)	172 (57.5–535.6)	0.07

Notes: ECOG: Eastern Cooperative Oncology Group; IQR: interquartile range.

**Table 2:** Therapeutic sequence and events potentially impacting survivals.

Variables	Study Population Study (*n* = 147)	Gemcitabine/Nab-Paclitaxel (*n* = 60)	FOLFIRINOX (*n* = 87)	*p*-Value
Number of resolved cases, median (IQR)	9 (5–12)	12 (9–18)	8 (5–10)	<0.001
Tumor response (RECIST), *n* (%)	31 (21.1%)	13 (21.7%)	18 (20.7%)	1
Management of patients within 4–6 months				
Best supportive care (in case of disease progression)	11 (7.5%)	7 (11.7%)	1 (1.1%)	1
Second-line therapy (in case of disease progression)	25 (17%)	10 (16.7%)	15 (17.2%)	1
Continuation treatment	28 (19%)	23 (38.3%)	5 (5.7%)	<0.001
Maintenance treatment	7 (4.8%)	1 (1.7%)	6 (6.9%)	0.24
Therapeutic break	4 (2.4%)	2 (3.3%)	2 (2.3%)	1
Consolidation chemoradiotherapy, *n* (%)	51 (34.7%)	7 (11.7%)	44 (50.6%)	<0.001
Secondary pancreatic resection, *n*(%)	24 (16.3%)	10 (16.7%)	14 (16.1%)	1
Duodenopancreatectomy	19 (79.2%)	8 (80%)	11 (78.6%)	1
Splenopancreatectomy	5 (20.8%)	2 (20%)	3 (21.4%)	
Pathological tumor				0.81
pT0	1 (4.5%)	0 (0%)	1 (7.7%)	
pT2	6 (27.3%)	3 (33.3%)	3 (23.1%)	
pT3	12 (54.5%)	5 (55.6%)	7 (53.8%)	
pT4	3 (13.6%)	1 (11.1%)	2 (15.4%)	
Pathological node				
pN+	11 (50%)	6 (66.7%)	5 (38.5%)	0.49
pN0	11 (50%)	3 (33.3%)	8 (61.5%)	0.10
pN1	9 (40.9%)	6 (66.7%)	3 (23.1%)	
pN2	2 (9.1%)	0 (0%)	2 (15.4%)	
Negative resection margin (R0)	16 (88.9%)	8 (88.9%)	12 (92.3%)	1
Grade 3–4 toxicity, *n* (%)	39 (27.6%)	16 (26.7%)	23 (28.4%)	0.97
Neutropenia	17 (11.6%)	11 (18.3%)	6 (6.9%)	0.062
Anemia	4 (2.7%)	4 (6.7%)	0 (0%)	0.054
Diarrhea	4 (2.7%)	2 (3.3%)	2 (2.3%)	1
Vomiting	4 (2.7%)	1 (1.7%)	3 (3.4%)	0.89
Second line of chemotherapy	65 (44.2%)	29 (48.3%)	36 (41.4%)	0.51
Gemcitabine			20 (23%)	
Gemcitabine/nab-paclitaxel			11 (12.6%)	
FOLFOX or XELOX		6 (10%)		
FOLFIRI		15 (25%)		
Nal-IRI (nanoliposomal irinotecan) LV5FU2		3 (5%)		
FOLFIRINOX		2 (3.3%)		
Other		3 (5.1%)	5 (5.7%)	
Third line of chemotherapy	19 (12.9%)	13 (21.7%)	6 (6.9%)	0.02

**Table 3:** Prognostic factors of the progression-free survival.

Prognostic Factor	Univariate Analyses		Multivariate Analyses (Adjusting for Age > 70 Years and ECOG)	
	*p*-Value	HR (95% CI)	*p*-Value	HR (95% CI)
Country				
France (*n* = 98) vs. Bulgaria (*n* = 12)	0.6	1.19 (0.62–2.30)		
Italy (*n* = 37) vs. Bulgaria (*n* = 12)	0.93	1.03 (0.50–2.13)		
Age (years)	0.6	0.99 (0.98–1.01)		
Age > 70 years (*n* = 45) vs. ≤70 years (*n* = 102)	0.83	0.96 (0.64–1.43)	0.12	0.55 (0.26–1.16)
Sex: male (*n* = 83) vs. female (*n* = 64)	0.58	1.1 (0.78–1.57)		
Arterial hypertension: no (*n* = 64) vs. yes (*n* = 51)	0.64	1.1 (0.74–1.64)		
Diabetes: no (*n* = 98) vs. yes (*n* = 49)	0.74	0.94 (0.65–1.36)		
History of myocardial ischemia: no (*n* = 105) vs. yes (*n* = 11)	0.83	0.93 (0.48–1.79)		
Performance status at the baseline: ECOG 1 (*n* = 60) vs. ECOG 0 (*n* = 73)	0.76	1.06 (0.73–1.54)	0.14	0.65 (0.36–1.15)
ECOG 2 (*n* = 7) vs. ECOG 0 (*n* = 73)	0.18	1.72 (0.78–3.78)	0.24	0.27 (0.03–2.38)
Abdominal pain: yes (*n* = 54) vs. no (*n* = 30)	0.027	1.8 (1.07–3.02)	0.035	2.03 (1.05–3.92)
Tumor location: head (*n* = 106) vs. tail (*n* = 7)	0.016	0.38 (0.17–0.84)	0.013	0.23 (0.07–0.73)
Body (*n* = 34) vs. tail (*n* = 7)	0.011	0.33 (0.14–0.78)	0.003	0.13 (0.03–0.5)
Tumor size (mm)	0.4	1.01 (0.99–1.02)		
Tumor size > 50 mm (*n* = 16) vs. ≤50 mm (*n* = 96)	0.21	1.45 (0.81–2.58)		
Bilirubin (µmol/L)	0.42	1 (1–1.01)		
Albumin (g/L)	0.89	1 (0.98–1.03)		
CA19-9 (UI/L)	0.28	1 (1–1)		
CA19-9 > 200 UI/L (*n* = 52) vs. ≤200 UI/L (*n* = 74)	<0.001	1.79 (1.22–2.63)	0.004	2.3 (1.3–4.08)
Tumor response (RECIST): yes (*n* = 35) vs. no (*n* = 110)	0.16	0.74 (0.48–1.12)		
Secondary surgery: yes (*n* = 24) vs. no (*n* = 123)	0.011	0.52 (0.32–0.86)	0.007	0.37 (0.18–0.76)
Chemotherapy regimen: FFX (*n* = 87) vs. GN (*n* = 60)	0.80	1.05 (0.73–1.51)	0.5	1.24 (0.66–2.32)

Notes: CI: confidence interval; ECOG: Eastern Cooperative Oncology Group; FFX: FOLFIRINOX; GN: gemcitabine/nab-paclitaxel; HR: hazard ratio; RECIST: response evaluation criteria in solid tumors.

**Table 4:** Prognostic factors for overall survival.

Prognostic Factor	Univariate Analyses		Multivariate Analyses (Adjusting for Age > 70 Years and ECOG)	
	*p*-Value	HR (95% CI)	*p*-Value	HR (95% CI)
Country				
France (*n* = 98) vs. Bulgaria (*n* = 12)	0.51	0.80 (0.41–1.56)		
Italy (*n* = 37) vs. Bulgaria (*n* = 12)	0.64	0.84 (0.40–1.74)		
Age (years)	0.85	1 (0.98–1.02)		
Age > 70 years (*n* = 45) vs. ≤70 years (*n* = 102)	0.60	1.12 (0.74–1.7)	0.92	1.03 (0.58–1.84)
Sex: male (*n* = 83) vs. female (*n* = 64)	0.42	1.16 (0.8–1.68)		
Arterial hypertension: no (*n* = 64) vs. yes (*n* = 51)	0.84	0.96 (0.64–1.44)		
Diabetes: no (*n* = 98) vs. yes (*n* = 49)	0.9	0.98 (0.66–1.44)		
History of myocardial ischemia: no (*n* = 105) vs. yes (*n* = 11)	0.31	0.71 (0.36–1.37)		
Performance status at the baseline: ECOG 1 (*n* = 60) vs. ECOG 0 (*n* = 73)	0.54	1.13 (0.77–1.66)	0.21	1.33 (0.85–2.08)
ECOG 2 (*n* = 7) vs. ECOG 0 (*n* = 73)	0.023	2.68 (1.14–6.28)	0.47	1.49 (0.51–4.36)
Abdominal pain: yes (*n* = 54) vs. no (*n* = 30)	0.1	1.55 (0.91–2.62)		
Tumor location: head (*n* = 106) vs. tail (*n* = 7)	0.042	0.42 (0.18–0.97)	0.1	0.45 (0.17–1.16)
Body (*n* = 34) vs. tail (*n* = 7)	0.033	0.38 (0.16–0.93)	0.032	0.32 (0.11–0.91)
Tumor size (mm)	0.27	1.01 (0.99–1.02)		
Tumor size > 50 mm (*n* = 16) vs. ≤50 mm (*n* = 96)	0.26	1.38 (0.79–2.42)		
Bilirubin (µmol/L)	0.4	1 (1–1.01)		
Albumin (g/L)	0.77	1 (0.98–1.03)		
CA19-9 (UI/L)	0.021	1 (1–1)		
CA19-9 > 200 UI/L (*n* = 52) vs. ≤200 UI/L (*n* = 74)	<0.001	1.8 (1.2–2.68)	0.015	1.71 (1.11–2.65)
Tumor response (RECIST): yes (*n* = 35) vs. no (*n* = 110)	0.19	0.74 (0.48–1.15)		
Secondary surgery: yes (*n* = 24) vs. no (*n* = 123)	0.002	0.4 (0.23–0.71)	0.003	0.38 (0.2–0.71)
Chemotherapy regimen: FFX (*n* = 87) vs. GN (*n* = 60)	0.70	1.08 (0.74–1.57)	0.60	1.15 (0.68–1.94)

Notes: CI: confidence interval; ECOG: Eastern Cooperative Oncology Group; FFX: FOLFIRINOX; GN: gemcitabine/nab-paclitaxel; HR: hazard ratio; RECIST: response evaluation criteria in solid tumors.

## Data Availability

The data presented in this study are available on request from the corresponding author Nicolas Williet.
